# Annotations

**Published:** 1895-10-05

**Authors:** 


### Annotations.

### The Classification of Paupers.

A subject has been brought before the North-Western Poor Law Conference by Dr. Milson Rhodes which deserves serious attention, for it is but the germ of a reform which must sooner or later go much further than he suggests. The basis of the Poor Law was originally the parish, but circumstances have changed, facilities of intercommunication have improved, and now it is obvious that in many cases the union itself is too small to form a fitting unit of Poor Law administration. While the conscience of the public is pricked by the hardships of the deserving poor, its sense of justice is outraged by the importunity and the laziness of the idle vagabond. Whatever else is done, a better classification of paupers is the first necessity. Yet in tiny workhouses, nay, even in larger unions, this classification is impossible. There is not space, nor is there convenience, for the many classes into which paupers should be divided; and above all things is this division difficult, owing to the fact that a different class of officer is required for the governance of each type of pauper. Some who are merely unfortunate deserve the greatest gentleness of treatment, while others who are vagabond and incorrigible require the severity of a martinet. What is required, then, is that the administrative area should be extended, that many unions should combine, and that, without spending money on new buildings, the workhouses already existing within each combination should be allocated each to its own special class of paupers. Clearly the time is ripe for a complete reorganisation of the Poor Law, and when that reorganisation takes form and shape it is to be hoped that as the union once superseded the older parish, so now will the County Council supersede the union as the administrative area.

### Sunstroke.

The recent wave of sultry weather, attended, as it has been, by a considerable number of cases of so-called sunstroke, has drawn attention afresh to the injurious effects of exposure to high temperatures. These take three main forms, only one of which can be correctly described as sunstroke. A large number of people, especially those who are elderly and whose hearts are feeble, suffer simply from the exhaustion due to the excessive heat. This syncopal form may end rapidly in death, but usually simple measures, such as removal to a cool place, frictions to the body, and cold to the head, will lead to restoration. True sunstroke, the direct effect of heat upon the nervous centres, comes on usually while exertion is being made, as in marching, under a blazing sun, especially when the air is hot and the clothing is inappropriate. The attack is often sudden, the patient becomes unconscious, both heart and respiration failing, and although under the influence of the cold douche recovery often takes place, such recovery is frequently imperfect; the mischief has resulted from an actual injury to the cerebro-spinal nervous system, an injury which is not confined to any one centre, and so we find in no small number of cases that serious impairment of health or intellect follows, indicative of structural changes having taken place in the brain. But in countries where the heat is continuously great the most common form of heat-stroke is that which occurs in houses, barracks, tents, ships, &c., away from the direct influence of the sun's rays. It comes on frequently in the night, especially among those who are exhausted by fatigue, or where there is overcrowding, and usually takes the form of a high fever, a condition of hyperpyrexia, with failure of heart and respiration, gasping breathing, restlessness, thirst, burning skin, livid or congested face, delirium, convulsions, and coma. This is a most serious condition, and first among the means of cure must be the lowering of the temperature. The damage, however, has been already done, and no amount of douching will restore the nervous system to its integrity; and so it happens that, in cases in which life is saved, the recovery is too often but imperfect, paralysis remaining, or the mind being left in a state of instability. In the occasional bursts of hot weather which we have in England, people should, of course, avoid exertion, especially in the sun's rays; but care about clothing in the day, and the air of bed-rooms in the night, are matters of, perhaps, even greater importance. Besides the few who suffer from sunstroke, a vast number suffer from heat, partly because they are swathed in tightly-fitting clothes, partly because they sleep in stifling bed-rooms filled with the hot air of the day, instead of the cool breezes of the night.

### The Death of M. Pasteur.

"France is losing all her great men." Such is the lament uttered by a newspaper correspondent over the death, all too early, of M. Pasteur. That is a sentiment which we cannot echo; but what we can feel is that a great man and an honourable is dead, and that the work and lessons of his life have been, and still are, of priceless value to mankind. M. Pasteur was a man of science, whose work, although purely scientific, almost invariably had practical ends in view. It was probably this practical bent of his mind, this desire to do something which should be of service to his fellow men, which made all his purely scientific work so immediately successful and so permanently enduring. "The germ theory"—what is it? It is a mere theory no longer. Pasteur has proved, and after him innumerable other persons have demonstrated for themselves, that the causes of fermentation and of putrefaction are living microbes, and the changes which take place as the result of their life-activity. What a whole universe of knowledge is here! How world-wide have been the practical applications of this knowledge! The brewer, the vine-grower, the silkworm cultivator, the farmer in all his grades, the physician, the surgeon—all have been put in possession of knowledge which has turned darkness into light, and ignorant incompetence into assured and successful skill. Pasteur was, in almost all the senses of the word, "a great man." He was a man whose work and methods constituted object-lessons for all other men, even for all other great men. If all men would but work as he worked, be truthful and simple as he was truthful and simple, be patient and untiring in their efforts at progress as he was patient and untiring, take nothing for granted until they could prove it, as he took nothing for granted without proof; above all, if they would be modest and avoid self-seeking, the whole world of science, morals, and practical business would be made anew. As a man of science, and as a great man in all the relations of life, civilisation, with France at its head, will mourn Pasteur.

